# The Differentiated Roles of Resilient Behavior and Job Crafting in Interaction with Work Intensity and Their Impact on Employee Health and Performance

**DOI:** 10.3390/ijerph22030429

**Published:** 2025-03-14

**Authors:** Amanda S. Voss, Roman Soucek, Klaus Moser, Hans Drexler

**Affiliations:** 1 Institute and Outpatient Clinic of Occupational, Social, and Environmental Medicine, Friedrich-Alexander-Universität Erlangen-Nürnberg (FAU), Henkestrasse 9-11, 91054 Erlangen, Germany; hans.drexler@fau.de; 2 School of Business, Economics and Society, Friedrich-Alexander-Universität Erlangen-Nürnberg (FAU), 90403 Nürnberg, Germany; roman.soucek@medicalschool-hamburg.de (R.S.); klaus.moser@fau.de (K.M.); 3 Department of Psychology, MSH Medical School Hamburg, 20457 Hamburg, Germany

**Keywords:** work intensity, work intensification, resilient behavior, job crafting, health, task performance

## Abstract

In recent years, changing working conditions have placed high demands on employees, resulting in increased work intensity, which may affect employees’ health and performance. Based on job and personal resources, individual behaviors help to cope with increased work intensity. We investigated two individual behaviors, namely resilient behavior, which is a reactive approach that helps to cope with adverse situations at work, and job crafting, which is a proactive approach to managing the constellation of job demands and job resources. In a study of 1108 employees, we used regression analyses to examine the interplay between work intensity and individual behaviors and their impact on various outcomes. Our results showed that resilient behavior moderated the relationship between work intensity and psychosomatic complaints. In addition, work intensity moderated the relationship between job crafting and task performance. In summary, individual behaviors can have different effects on the constellation of work intensity and specific outcomes. Our findings on the specific effects of resilient behavior and job crafting allow for a more targeted application. Since the above behaviors can be enhanced through training, organizations can embrace this idea to maintain employees’ health and performance.

## 1. Introduction

The nature of work has changed significantly in recent years, driven by globalization, technological advances, and increasing demands for flexibility [1]. Digitalization has led to new ways of working, such as remote working and flexible working hours. This has been accompanied by an intensification in work, characterized by higher job demands and performance expectations [2], which has created new challenges for employees, leading to increased stress and making it more difficult to reconcile work and private life. Work intensity can increase productivity in the short term, but prolonged exposure is associated with negative outcomes such as burnout and reduced well-being [3]. In addition, high work intensity is associated with negative health outcomes, including psychosomatic complaints and lower life satisfaction [4,5]. Given these risks, it is important to understand how organizations and employees can effectively manage high work intensity.

The Job Demands–Resources (JD-R) model provides a useful framework for understanding how job demands and job resources interact to influence employee well-being and performance [6]. This model was inspired by Karasek’s Job Strain model [7], which examines the relationships between job demands, job decision latitude, and mental strain. Decision latitude has been incorporated into the JD-R model, where it is listed alongside other job resources such as social support and performance feedback [8]. Specifically, the JD-R model describes two processes in which job demands lead to strain and job resources promote motivation [8]. Ideally, job resources counteract the emergence of high work intensity as a job demand. For example, Soucek et al. [9] showed that job resources, such as autonomy, can help to reduce the degree of work intensity resulting from flexibility demands. Later versions of the JD-R model introduced personal resources [8]. However, both job and personal resources do not counteract job demands on their own but are rather antecedents of individual behaviors. These behaviors, in turn, enable coping with job demands, thereby ensuring well-being and promoting performance. We examine the effects of two specific individual behaviors, namely resilient behavior and job crafting. Resilient behavior follows a reactive approach that describes coping successfully with adverse situations at work, whereas job crafting describes a proactive approach that shapes the constellation of job resources and job demands.

The purpose of this study is to examine how individual behaviors contribute to employee health and performance in the context of high work intensity. Specifically, it examines whether resilient behavior can buffer the negative health effects of high work intensity and how work intensity affects the relationship between job crafting and task performance. By examining these relationships, this study contributes to a better understanding of how employees can sustain their well-being and performance in demanding work environments. The findings have practical implications for organizations seeking to support employees through targeted interventions, such as resilience training and job crafting workshops, to mitigate the negative effects of work intensity.

### 1.1. Work Intensity

Work intensity refers to the degree to which employees are required to maintain high levels of effort and performance over time [10,11]. However, work intensity is a multidimensional construct that goes beyond “working hard”. Piasna [12] argued that work intensity cannot be fully understood simply as working at high speeds under tight deadlines, as these aspects do not capture the full complexity of modern work dynamics. The flexibility of modern work arrangements has introduced new forms of work that not only increase the quantity and pace of tasks but also introduce qualitative changes that contribute to work intensity. In this vein, Soucek and Voss [13] identified seven facets of work intensity that encompass both quantitative and qualitative aspects. This broad conceptualization of work intensity addresses the need for employees to be more proactive in managing their work schedules, planning and prioritizing tasks, and navigating the blurred boundaries between work and personal life—especially in the context of remote work. A growing body of literature on work intensity suggests that it plays an important role in employees’ mental health, with some studies focusing on the consequences of intense work environments [14], while others see work intensity as a precursor to health problems [4].

### 1.2. Work Intensity and Its Negative Consequences

High work intensity is often associated with increased stress, emotional exhaustion, and health impairment, as employees may have difficulty recovering adequately from continuous work demands [3]. In addition, intensified work conditions can undermine job satisfaction and long-term performance, especially in environments with insufficient or inadequate job resources [2]. Another consequence is irritation, which reflects a state of mental impairment caused by perceived discrepancies between goals and outcomes [15]. This irritation can manifest both cognitively (e.g., rumination or inability to disengage from work-related thoughts) and emotionally (e.g., irritability or anger). Emotional irritation has been associated with symptoms leading to burnout or depression [15]. Furthermore, employees exposed to high work intensity often experience adverse health effects, including increased psychosomatic complaints [3]. Given these negative consequences of work intensity, we hypothesize that work intensity is negatively related to psychosomatic complaints.

**Hypothesis** **1.** *Work intensity is negatively related to psychosomatic complaints*.

### 1.3. The Interplay of Work Intensity and Individual Behaviors

According to the JD-R model, job and personal resources can buffer the negative effects of high job demands [8]. Traditionally, job resources such as social support or autonomy have been emphasized as critical factors in mitigating work-related stress. However, some studies have emphasized the role of personal resources, i.e., individual characteristics and skills, that enable employees to cope with demanding work conditions [16]. Because job and personal resources are manifested in individual behaviors, we expect similar mechanisms to operate. In particular, we examine resilient behavior and job crafting as individual behaviors and their influence on the relationship between work intensity and various outcomes. However, not all individual behaviors have the same effect. Because they are nourished by resources, they can contribute directly to the motivational process in the JD-R model or act as a buffer of the strain process. In particular, we expect resilient behavior to moderate the relationship between work intensity and health outcomes. In contrast, we expect job crafting to contribute directly to task performance. Furthermore, we expect a moderating effect of work intensity on the relationship between job crafting and task performance.

#### 1.3.1. Resilient Behavior

Resilient behavior is a reactive mechanism that enables employees to recover from adversity and maintain stability in challenging work conditions [17]. It helps employees manage stress and reduce psychosomatic complaints by promoting adaptive coping strategies [18]. Resilient individuals recover quickly from setbacks and stress, can adapt well to challenging work conditions, and grow from challenges. Resilient behavior describes various strategies for self-regulation and includes both emotion- and problem-oriented coping strategies [19]. Some studies suggest that resilience acts as a stress buffer, mitigating the detrimental effects of high work demands on health outcomes [20,21,22]. For example, resilience enables healthcare professionals to cope with high levels of effort and reduces emotional exhaustion [21], or resilience buffers the relationship between verbal mental demands and work overload [22]. Therefore, we hypothesize that resilient behavior attenuates the effect of work intensity on psychosomatic complaints.

**Hypothesis** **2.** *Resilient behavior weakens the relationship between work intensity and psychosomatic complaints.*

#### 1.3.2. Job Crafting

Job crafting represents a proactive approach in which employees modify their work tasks, work relationships, or cognitive framing of their work to optimize person–job fit [23]. This self-initiated behavior can increase engagement and task performance by aligning work tasks with individual strengths and preferences [24]. Research suggests that employees who engage in job crafting tend to experience higher job satisfaction and task performance because they can shape their work environment to better meet their needs [25]. Accordingly, we hypothesize that job crafting increases task performance.

**Hypothesis** **3.** *Job crafting is positively related to task performance.*

Employees who engage in job crafting are likely to maintain higher task performance, even under high work intensity, by actively shaping their work environment. However, work intensity can be a limitation to job crafting. While proactive job crafting can enhance performance, extreme work intensity may limit employees’ ability to effectively change their tasks and responsibilities [25]. Given that work intensity is a hindering demand [26], we hypothesize that work intensity weakens the positive relationship between job crafting and task performance.

**Hypothesis** **4.** *Work intensity weakens the positive relationship between job crafting and task performance.*

**Figure 1:**
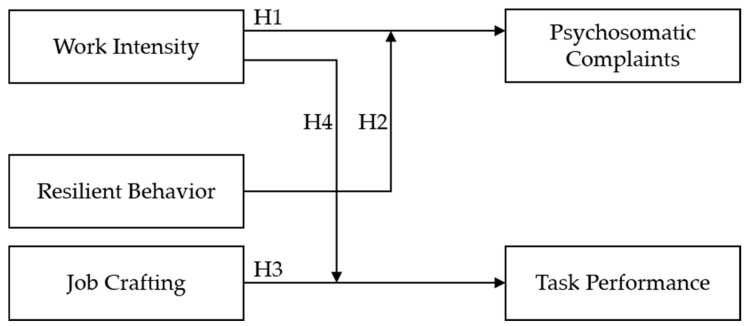
Overview of hypotheses.

Figure 1 provides an overview of the hypotheses.

## 2. Materials and Methods

In this study, we used a cross-sectional survey design to examine the role of individual behaviors in the relationship between work intensity and employee outcomes. Specifically, we used regression analyses to examine how resilient behavior moderates the effects of work intensity on psychosomatic complaints and how work intensity moderates the relationship between job crafting and task performance. The sample consisted of employees from a public administration organization who completed a web-based survey.

All procedures complied with the ethical standards of the institutional and national research committee and with the 1964 Helsinki Declaration and its later amendments. The study was approved by the Ethics Commission of the School of Business, Economics, and Society at the Friedrich-Alexander-Universität Erlangen-Nürnberg (28 September 2020). Informed consent for participation was obtained from all subjects involved in this study.

### 2.1. Sample

The survey was completed by 1108 employees, representing a participation rate of 34% of all employees in the organization. Of the respondents, 22% identified as male, and the mean age of the participants was 45.91 years (SD = 11.94). The sample included employees working under a variety of conditions, with 15% working from home, and 14% working from any location. On average, participants worked 36.56 h per week (SD = 6.19). Supervisory roles were held by 19% of the participants. The sample was broadly representative of the organization’s workforce in terms of demographic characteristics when compared to the company’s organizational records. However, the proportion of employees in supervisory roles was higher in the sample than in the overall workforce, which led us to include supervisory position as a control variable, along with other demographic factors.

### 2.2. Measures

#### 2.2.1. Work Intensity

Work intensity was assessed using the short version of the WI-7 scale [27], a validated instrument that assesses both quantitative and qualitative dimensions of work intensity with 7 items. The scale measures aspects such as time pressure, task volume, multitasking, role ambiguity, and the blurring of work–life boundaries. An example item is as follows: “I have a lot of tasks to do”. Responses were collected on a 5-point Likert scale ranging from 1 (*does not apply at all*) to 5 (*fully applies*). Internal consistency was acceptable; Cronbach’s alpha was α = 0.75.

#### 2.2.2. Psychosomatic Complaints

Psychosomatic complaints were assessed using a validated 20-item scale that captures symptoms commonly associated with work-related stress, such as headaches, back pain, sleep disturbances, and gastrointestinal problems [28]. Responses are recorded on a 5-point Likert scale, ranging from 1 (*never*) to 5 (*almost daily*). An example item is as follows: “Do you tire easily?”. Internal consistency was excellent; Cronbach’s alpha was α = 0.92.

#### 2.2.3. Task Performance

Task performance was measured by a 5-item self-report assessment of behaviors that are recognized by formal reward systems and are part of the requirements as described in job descriptions [29]. Participants rated their ability to meet deadlines, manage workload, and complete tasks in a timely manner on a 7-point Likert scale ranging from 1 (*very poorly*) to 7 (*very well*). An example item is as follows: “I perform assigned job duties appropriately”. Internal consistency was high; Cronbach’s alpha was α = 0.83.

#### 2.2.4. Resilient Behavior

Resilient behavior in the workplace was assessed using the short version of the questionnaire by Soucek et al. [19]. This questionnaire consists of eight items describing different behavioral responses to adverse events at work (e.g., “Even in critical situations at work, I can cope with my anger”) and asks participants to indicate their agreement on a 7-point scale ranging from 1 (*does not apply at all*) to 7 (*applies completely*). Internal consistency was strong; Cronbach’s alpha was α = 0.86.

#### 2.2.5. Job Crafting

Job crafting was measured using a validated instrument [30] with 15 items. We assessed the subscales of increasing structural job resources, increasing social job resources, and increasingly challenging job demands. Participants indicated their answers on a 5-point Likert scale ranging from 1 (*does not apply at all*) to 5 (*fully applies*). An example item is as follows: “I try to develop my capabilities”. Internal consistency was acceptable; Cronbach’s alpha was α = 0.79.

#### 2.2.6. Control Variables

To account for potential confounding factors, several control variables were included in the analysis. We asked participants about demographic and work-related variables that have been associated with work intensity [9,11]. The control variables were age, gender, supervisory position, working from home (i.e., having a fully equipped workspace at home), working from anywhere (i.e., working in changing locations outside of the company), and working hours. Those who worked from home had a fully equipped workplace in their home office, while those who worked from anywhere did not have a fully equipped workplace (i.e., they only had a laptop for mobile work in different locations). This difference was due to different contractual arrangements. We also included decision latitude as a control variable because it has been shown to be associated with work intensity [9].

Gender was dummy coded as 0 (*female*) and 1 (*male*). Participants reported their age in years and the number of hours they worked per week. Supervisory position, working from home, and working from anywhere were dummy coded as 0 (*no*) and 1 (*yes*). Decision latitude was assessed using three items on a 5-point Likert scale ranging from 1 (*very little*) to 5 (*very much*) [31]. An example item is as follows: “Can you plan and schedule your work independently?” Cronbach’s alpha was α = 0.65.

### 2.3. Analyses

Hierarchical regression analyses were used to test the hypotheses. All models were tested using the statistical software R, version 4.4.2 [32]. Moderated regression analyses were conducted to examine the moderating effect of resilient behavior on the relationship between work intensity and psychosomatic complaints and to examine the moderating effect of work intensity on the relationship between job crafting and task performance. Data and materials are available upon request from the corresponding author.

## 3. Results

Table 1 shows the descriptive statistics and correlations between the study variables. Work intensity was significantly correlated with psychosomatic complaints (*r* = 0.21, *p* < 0.001) and task performance (*r* = −0.15, *p* < 0.001). In addition, work intensity was associated with both resilient behavior (*r* = −0.08, *p* = 0.009) and job crafting (*r* = 0.21, *p* < 0.001). Male gender was negatively correlated with psychosomatic complaints (*r* = −0.25, *p* < 0.001), accompanied by younger age (*r* = −0.08, *p* = 0.012), more frequent supervisory position (*r* = 0.09, *p* = 0.002), and higher resilient behavior (*r* = 0.10, *p* = 0.001).

### 3.1. Work Intensity and Health

Table 2 summarizes the hierarchical regression analyses for predicting psychosomatic complaints. Model 1a included the control variables of gender, age, supervisory position, working from home, working from anywhere, hours worked per week, and decision latitude. Among these, male participants had lower levels of psychosomatic complaints (*b* = −0.42, *p* < 0.001), which was also true for participants who reported a high decision latitude (*b* = −0.11, *p* < 0.001). Model 1b additionally included work intensity, which was positively associated with psychosomatic complaints (*b* = 0.36, *p* < 0.001), confirming Hypothesis 1.

### 3.2. Moderating Effect of Resilient Behavior

**Figure 2:**
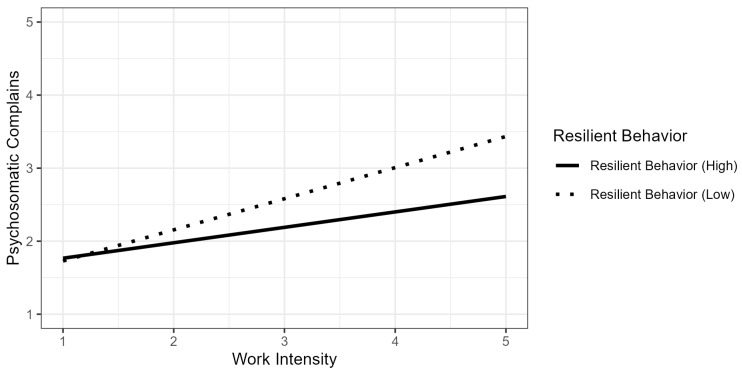
Interaction effect of work intensity and resilient behavior on psychosomatic complains.

In Table 2, Model 1c included the main effects of individual behaviors. While resilient behavior had a negative effect (*b* = −0.24, *p* < 0.001), the results showed no effect of job crafting (*b* = 0.02, *p* = 0.656). Model 1d added the interaction effects. The results show that resilient behavior moderated the relationship between work intensity and psychosomatic complaints (*b* = −0.12, *p* = 0.001), such that resilient behavior weakened the positive association between work intensity and psychosomatic complaints, confirming Hypothesis 2. This interaction effect is shown in Figure 2, which indicates that the effect of work intensity on psychosomatic complaints is weaker when resilient behavior is high.

### 3.3. Job Crafting and Task Performance

Table 3 summarizes the hierarchical regression analyses for predicting task performance. Model 2a included the control variables of gender, age, supervisory position, working from home, working from anywhere, weekly hours worked, and decision latitude. Model 2b added work intensity as a predictor, which was negatively related to task performance (*b* = −0.16, *p* < 0.001). Model 2c expanded the predictors to include individual behaviors, both of which had a positive effect on task performance, resilient behavior (*b* = 0.24, *p* < 0.001), and job crafting (*b* = 0.11, *p* = 0.006).

**Figure 3:**
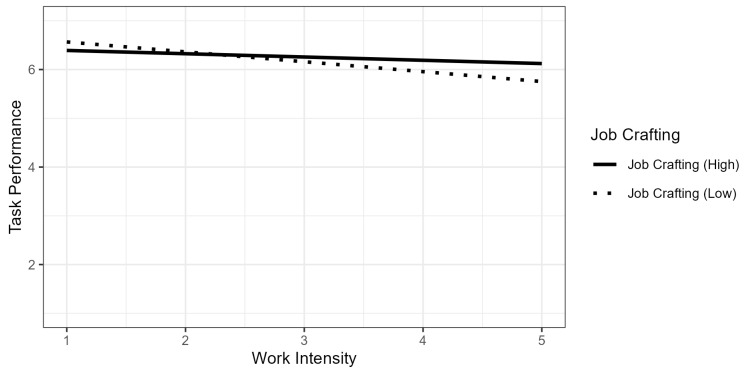
Interaction effect of work intensity and job crafting on task performance.

Finally, Model 2d added the moderating effects of work intensity and individual behaviors. Work intensity moderated the relationship between resilient behavior and task performance (*b* = 0.08, *p* = 0.013). In addition, work intensity also strengthened the effect of job crafting on task performance (*b* = 0.13, *p* = 0.012). Hypothesis 4 was rejected. The interaction effect is presented in Figure 3, indicating that work intensity has a negative effect on task performance in the case of low job crafting.

## 4. Discussion

In summary, our study highlights that individual behaviors such as resilient behavior and job crafting can buffer the negative effects of high work intensity on both psychosomatic complaints and task performance. Our findings suggest that work intensity plays a crucial role in explaining how employees’ mental health and performance are affected by job demands. Specifically, we found that work intensity was positively related to psychosomatic complaints and that resilient behavior moderated this relationship. Individuals who exhibit higher levels of resilient behavior are better able to cope with the negative effects of high work intensity, resulting in fewer psychosomatic complaints. However, job crafting did not have a similar buffering effect on psychosomatic complaints. One possible explanation is that job crafting functions primarily as a motivational strategy to optimize work tasks rather than as a coping mechanism for psychological distress. Accordingly, we found a direct effect of job crafting on task performance. Work intensity moderated this relationship between job crafting and task performance, strengthening the correlation between job crafting and task performance. This surprising result may indicate that work intensity in this case acts as a challenging demand rather than a hindering demand. Future research should further investigate whether different forms of job crafting (e.g., increasing structural job resources vs. reducing job demands) may have different effects on health and performance outcomes.

These findings underscore the importance of individual behaviors in mitigating the negative consequences of high work intensity. Resilient behavior appears to act as a protective factor against both health outcomes, consistent with previous research emphasizing the role of resilience in buffering stress [21,22]. The concept of resilient behavior helps to bridge the gap between personal resources and psychological well-being [19]. More specifically, the buffer hypothesis suggests that personal resources can help employees by enhancing their ability to cope with demanding work conditions [8,16]. Although this hypothesis has been explored in various contexts, the specific mechanisms remain poorly understood. However, Soucek et al. [19] showed that resilient behavior contributed to psychological well-being over and above the influence of demographic variables and personal resources.

Similarly, job crafting, as a proactive strategy, appears to enable employees to better manage the demands of their jobs, thereby maintaining performance even under stressful conditions [33]. While resilient behavior represents a reactive mechanism that enables individuals to withstand stress and recover from adversity, job crafting is consistent with proactive self-regulation strategies. According to self-regulation theories, individuals actively shape their work environments to align with personal goals and well-being, rather than simply reacting to existing stressors. Parker and Bindl [34] emphasized that proactive motivation plays a key role in shaping job characteristics, which is consistent with our classification of job crafting and resilient behavior as individual behaviors that enable individuals to modify and cope with job demands in ways that support performance and well-being. This distinction highlights the need to consider both reactive and proactive strategies when designing workplace interventions aimed at mitigating the negative effects of high work intensity.

Within the JD-R model [8], job crafting is positioned separately from job and personal resources, although one could argue that the ability to do job crafting is itself a resource. However, this construct shows very well that it depends on the availability of resources, such as flexibility within the job, but also on individual abilities such as self-regulation and knowing how to craft the job in a favorable way. With our study, we want to emphasize that it is not only job crafting as a proactive behavior that is an important bridge between resources and outcomes, but this also applies to other resources such as resilient behavior. From an organizational and scientific perspective, it is important to observe if and how resources are translated into actions.

### 4.1. Limitations

This study has several limitations. First, the cross-sectional design prevents us from making causal inference conclusions about the relationships between work intensity, individual behaviors, and outcomes such as task performance and psychosomatic complaints. Future research should use longitudinal designs to examine how these relationships evolve over time. Second, the use of self-reported data raises concerns about common method bias [35]. Future studies should consider using multiple sources of data, such as supervisor ratings or physiological measures, to increase validity. In addition, potential self-selection effects should be considered: Employees with higher resilient behavior might already be more likely to take on demanding work environments, which may have influenced our findings. Third, our sample was limited to a specific group of employees, and future research should examine these findings in other industries and more diverse populations to confirm the generalizability of the results.

### 4.2. Implications

From a theoretical perspective, these two individual behaviors need to be conceptually integrated into the JD-R model. An extended version of the model [8] follows a dynamic approach and describes loss and gain spirals driven by individual behaviors. Loss spirals result from self-undermining as a vicious cycle of high job demands and strain. Gain spirals of resources and work engagement are created by engaged employees through job crafting. We believe that the two individual behaviors can be aligned with these spirals. Resilient behavior describes successfully coping with adverse situations, thus breaking the loss spiral between job demands and strain. Job crafting describes the proactive management of the constellation of job resources and job demands and should therefore contribute to the gain spiral between job resources and motivation. Since we found gender differences with lower levels of psychosomatic complaints for men, which were accompanied by other structural differences and higher levels of resilient behavior, future research should investigate whether differences in the use of individual behaviors are due to gender per se or related to job characteristics.

Our study aims to provide a more nuanced understanding of how individual behaviors can mitigate the negative effects of high work intensity. The findings have practical implications for organizations seeking to support employees through targeted interventions, such as resilience training and job crafting workshops, to maintain both health and performance in demanding work environments. Specifically, resilience-building interventions, such as cognitive–behavioral training or stress management workshops, can help employees develop adaptive coping strategies [36]. In addition, organizations should promote job crafting not only through individual training but also by designing work environments that allow employees greater autonomy and flexibility in the design of their tasks. For instance, leaders can actively support job crafting by providing structured feedback, encouraging role innovation, and creating collaborative workspaces that facilitate proactive task adaptation [23]. Finally, organizations should consider how to design work environments that foster these behaviors, as well as encourage employees to engage in strategies that mitigate the negative effects of work intensity while maintaining performance levels.

## 5. Conclusions

This research highlights the importance of individual behaviors in promoting a healthier, more productive workforce in the face of increasing job demands. Specifically, this study showed that work intensity has a significant impact on psychosomatic complaints, but reactive behaviors such as resilient behavior can effectively buffer these effects. In addition, proactive behaviors such as job crafting contribute to task performance. In addition to providing the necessary job resources, organizations should also focus on supporting individual behaviors to help employees cope with high work intensity and improve both their well-being and performance.

## Figures and Tables

**Table 1:** Descriptive statistics and correlations between study variables.

		M	SD	1	2	3	4	5	6	7	8	9	10	11
1	Gender ^a^	0.22	0.41											
2	Age	45.91	11.94	−0.08 *										
3	Supervisory position ^b^	0.19	0.39	0.09 **	0.17 ***									
4	Working from home ^b^	0.15	0.36	−0.01	0.01	−0.06 *								
5	Working from anywhere ^b^	0.14	0.35	0.15 ***	0.03	0.12 ***	−0.17 ***							
6	Hours worked	36.56	6.19	0.18 ***	−0.13 ***	0.11 ***	0.06 *	0.06 *						
7	Decision latitude	3.21	0.81	0.06 *	0.09 **	0.20 ***	0.13 ***	0.20 ***	0.08 **					
8	Work intensity	3.07	0.61	0.04	−0.07 *	0.24 ***	0.04	0.21 ***	0.10 ***	0.10 ***				
9	Resilient behavior	5.04	0.87	0.10 **	−0.02	0.07 *	0.00	0.06	0.03	0.13 ***	−0.08 **			
10	Job crafting	3.09	0.51	0.05	−0.27 ***	0.10 ***	0.01	0.13 ***	0.08 **	0.23 ***	0.21 ***	0.43 ***		
11	Psychosomatic complains	2.41	0.78	−0.25 ***	0.08 **	−0.04	−0.01	−0.09 **	−0.11 ***	−0.13 ***	0.21 ***	−0.32 ***	−0.12 ***	
12	Task performance	6.20	0.62	−0.07 *	0.02	−0.04	0.04	−0.03	−0.08 **	0.04	−0.15 ***	0.37 ***	0.19 ***	−0.14 ***

*Notes*. *N* = 1108; ^a^ gender was dummy coded as 0 (*female*) and 1 (*male*); ^b^ dummy coded as 0 (*no*) and 1 (*yes*); * *p* < 0.05; ** *p* < 0.01; *** *p* < 0.001.

**Table 2:** Hierarchical regression results for psychosomatic complains.

	Model 1a	Model 1b	Model 1c	Model 1d
Intercept	2.91 ***	1.83 ***	3.01 ***	1.51 *
Gender ^a^	−0.42 ***	−0.40 ***	−0.36 ***	−0.36 ***
Age	0.00 *	0.01 ***	0.01 **	0.01 **
Supervisory position ^b^	0.01	−0.12 *	−0.09	−0.08
Working from home ^b^	0.00	−0.05	−0.05	−0.05
Working from anywhere ^b^	−0.08	−0.21 **	−0.18 **	−0.17 **
Hours worked	−0.01	−0.01 *	−0.01 *	−0.01 *
Decision latitude	−0.11 ***	−0.11 ***	−0.09 **	−0.09 **
Work intensity		0.36 ***	0.32 ***	0.79 ***
Resilient behavior			−0.24 ***	0.15
Job crafting			0.02	−0.12
Work intensity × Resilient behavior				−0.12 **
Work intensity × Job crafting				0.05
*R* ^2^	0.08	0.16	0.22	0.23
Δ*R*^2^		0.07 ***	0.06 ***	0.01 **

*Notes*. *N* = 1108; unstandardized regression coefficients; ^a^ gender was dummy coded as 0 (*female*) and 1 (*male*); ^b^ dummy coded as 0 (*no*) and 1 (*yes*); * *p* < 0.05; ** *p* < 0.01; *** *p* < 0.001.

**Table 3:** Hierarchical regression results for task performance.

	Model 2a	Model 2b	Model 2c	Model 2d
Intercept	6.31 ***	6.80 ***	5.22 ***	7.77 ***
Gender ^a^	−0.07	−0.08	−0.12 **	−0.12 **
Age	0.00	−0.00	0.00	0.00
Supervisory position ^b^	−0.06	−0.00	−0.05	−0.05
Working from home ^b^	0.06	0.08	0.08	0.08
Working from anywhere ^b^	−0.03	0.02	−0.01	−0.04
Hours worked	−0.01 *	−0.01 *	−0.01 *	−0.01 *
Decision latitude	0.05	0.05	0.00	0.01
Work intensity		−0.16 ***	−0.13 ***	−0.94 ***
Resilient behavior			0.24 ***	−0.01
Job crafting			0.11 **	−0.31
Work intensity × Resilient behavior				0.08 *
Work intensity × Job crafting				0.13 *
*R* ^2^	0.02	0.04	0.18	0.19
Δ*R*^2^		0.02 ***	0.14 ***	0.02 ***

*Notes*. *N* = 1108; unstandardized regression coefficients; ^a^ gender was dummy coded as 0 (*female*) and 1 (*male*); ^b^ dummy coded as 0 (*no*) and 1 (*yes*); * *p* < 0.05; ** *p* < 0.01; *** *p* < 0.001.

## Data Availability

The data presented in this study are available on request from the corresponding author.
